# New cine magnetic resonance imaging parameters for the differential diagnosis of chronic intestinal pseudo-obstruction

**DOI:** 10.1038/s41598-021-02268-1

**Published:** 2021-11-26

**Authors:** Hiroki Sato, Hiroyuki Ogihara, Kazuya Takahashi, Yuzo Kawata, Yuichi Kojima, Kentaro Tominaga, Junji Yokoyama, Yoshihiko Hamamoto, Shuji Terai

**Affiliations:** 1 Division of Gastroenterology, Graduate School of Medical and Dental Sciences, Niigata University, 757-1, Asahimachidori, Chuo-ku, Niigata-city, Niigata 951-8510 Japan; 2 Division of Electrical, Electronic, and Information Engineering, Yamaguchi University Graduate School of Sciences and Technology for Innovation, Yamaguchi, 755-8611 Japan

**Keywords:** Gastroenterology, Gastrointestinal diseases

## Abstract

Chronic intestinal pseudo-obstruction (CIPO) is a severe and refractory intestinal motility disorder whose diagnosis currently relies on subjective imaging assessments. Cine magnetic resonance imaging (MRI) may potentially improve the quantitative analysis of gastrointestinal motility; however, suitable CIPO detection parameters should be determined. Cine MRI was performed in seven patients with CIPO and 11 healthy controls. The logarithm of the Mahalanobis distance (x_1_) and distance variation per time (x_2_) were used as the original parameters to determine CIPO diagnostic thresholds. Furthermore, the correlation between cine MRI findings and CIPO severity was investigated. Threshold values of α = 1.10 and β = 0.15 for x_1_ and x_2_, respectively, produced a CIPO diagnosis sensitivity of 1.00 (7/7) and specificity of 0.82 (9/11). The resulting error was 0.11 (2/18). The two parameters were correlated (Pearson’s correlation coefficient: − 0.52). Any of the intestinal tracts of patients with severe CIPO requiring home parenteral nutrition belonged to the region defined by x_1_ ≥ 1.10 and x_2_ ≤ 0.15. Cine MRI is effective for the quantitative evaluation of small intestinal motility and CIPO diagnosis when using the abovementioned parameters and can be useful for treatment decision-making. However, these parameters have a wide distribution in healthy volunteers; this may complicate the detection of other disorders.

## Introduction

Chronic intestinal pseudo-obstruction (CIPO) is a rare refractory disorder of the intestine^1^ wherein intestinal motility is severely impaired. It causes patients to experience abdominal pain, distention, vomiting, and weight loss despite the absence of organic disorders in the intestinal lumen. Recurrent paralytic ileus is a complication that causes social discomfort and necessitates repeated hospitalization for intestinal tract decompression or (at least) bowel rest. Furthermore, intestinal strangulation requires surgical resection of the necrotic segment, as that is the only strategy that can save the patient’s life. However, patients who require resection of the intestine risk developing adhesive ileus or short bowel syndrome when extensive resection of the small intestine is performed. If their symptoms become more severe owing to intestinal motility failure, or malabsorption occurs owing to short bowel syndrome, home parenteral nutrition (HPN) becomes necessary to supplement nourishment^2^.

Histological examination of the impaired intestine is a common approach to investigate CIPO of unknown etiology. Based on previously reported patients, CIPO is considered a collection of several pathologies that include neuropathy, abnormalities of intestinal cells of Cajal, and myopathies^3–6^. However, in most cases, histological investigation does not yield a diagnosis, suggesting that CIPO has molecularly heterogeneous underlying causes. Furthermore, researchers are able to obtain specimens only from patients with advanced disease given that obtaining samples for histology will not influence treatment decision-making, and such procedures may worsen the underlying dysmotility (as mentioned above).

**Figure 1:**
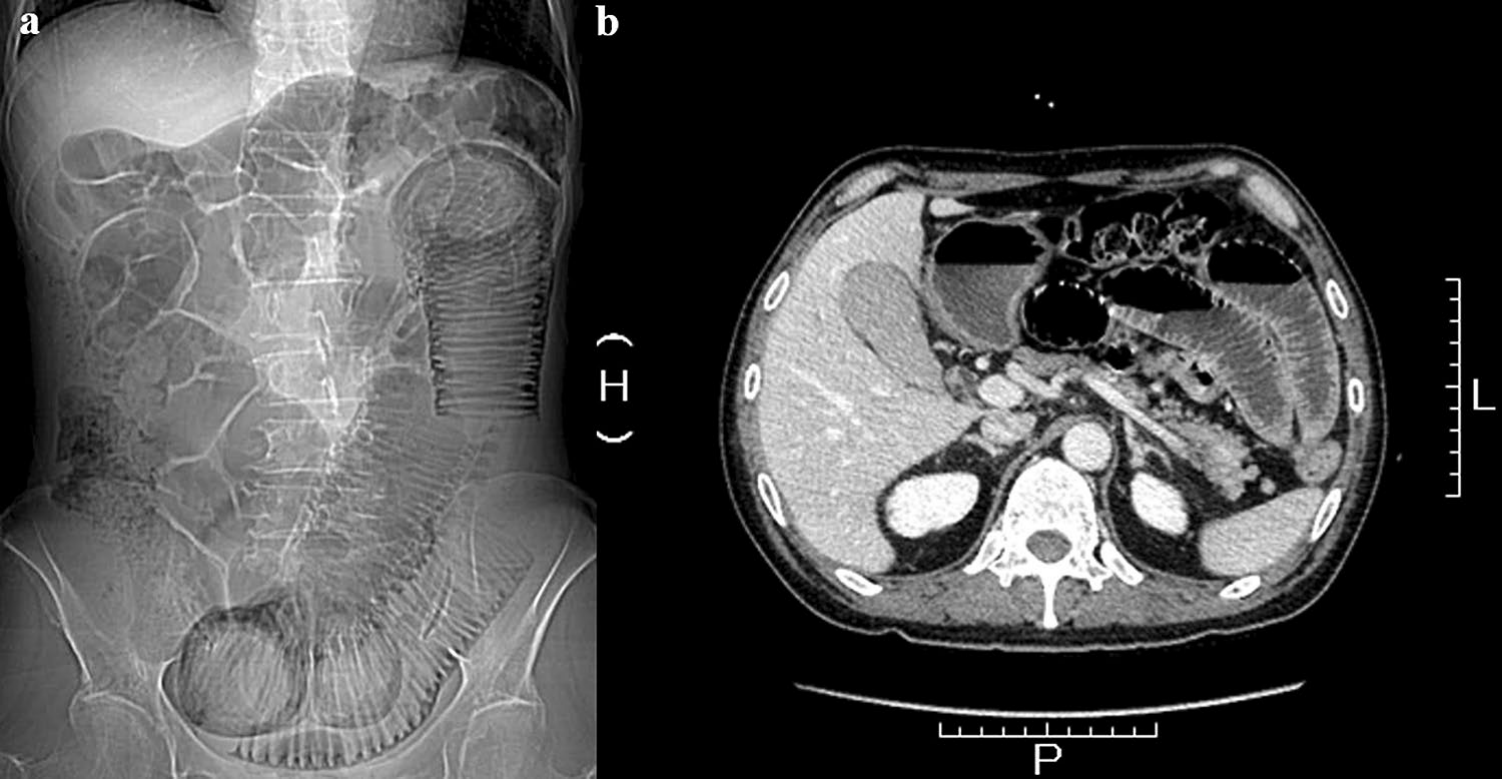
Typical radiological images of chronic intestinal pseudo-obstruction including plain radiograph (**a**) and computed tomography (**b**).

Histological investigation can only be performed for critical patients and the subsequent clinical course does not necessarily improve (and can even worsen in some cases); therefore, radiological imaging is preferable for diagnosing CIPO. In typical cases, non-obstructive ileus findings are observed on radiography and computed tomography (Fig. 1). However, these modalities carry a risk of radiation exposure; moreover, radiography is not sufficiently sensitive or specific to detect ileus, particularly in its early stages when symptoms are relatively light and/or the duration since disease onset has been relatively short^1^. Therefore, other medical methodologies for the quantitative analysis of intestinal motility are recommended for CIPO diagnosis. Several modalities have been reported to be suitable for this purpose, although none are considered a gold standard owing to varying advantages and disadvantages^7–9^. For example, intestinal manometry can analyze the peristaltic wave at a certain point; however, the limited length of the catheter makes it impossible to analyze the distal parts of the intestine. Small bowel transit time on capsule endoscopy has intra-and inter-individual discrepancies^10^, and capsule endoscopy carries the risk of retention and is contraindicated in patients with CIPO. On the other hand, recent advances in magnetic resonance imaging (MRI) have led to the development of cine MRI, a noninvasive radiation-free tool to assess organ function and diagnose certain diseases, particularly for cardiovascular conditions^11^. As such, cine MRI can be applicable to gastrointestinal disorders, including CIPO^12,13^, and to establish parameters for cine MRI readings is an unmet requirement in CIPO^14^. Moreover, the inter- and intra-observer discrepancy remains a hindrance for objective diagnosis.

Therefore, the aim of this study was to establish new parameters for cine MRI that would assist in the diagnosis of CIPO.

## Methods

### Cine MRI protocol

CIPO was diagnosed in cases having long-term persistent abdominal symptoms for more than 6 months, no mechanical cause, bowel dilatation with air-fluid levels in radiological images^1^. After 8 h of fasting, the participants underwent MRI in the supine position after orally ingesting 1000 mL of water to fill the small intestine. Patients who were unable to ingest the entire amount because of severe abdominal symptoms were encouraged to drink as much as possible to prevent worsening of symptoms^13^ .

Imaging was performed using a 1.5-T MRI unit (SIGNA™ Creator, GE Healthcare, USA). Before performing cine MRI, coronal images of the entire abdomen were obtained to identify an appropriate imaging plane. A steady-state free precession sequence (FIESTA sequence: repetition time = 3.6 ms, echo time = 1.6 ms, flip angle = 70°, slice thickness = 10 mm, matrix = 196 × 230, field of view = 400 × 400 mm, number of excitations = 1.0, bandwidth = 100 kHz, and fat suppression = special [inversion time = 200 ms]) was used for imaging, which allowed for continuous scanning without intervals for each image. Three slice location were separately acquired. For each slice location, sequential scanning was performed every 0.571 s with 35 images. The patients held their breaths for 20 s.

All procedures involving human participants were performed in accordance with the principles of the 1964 Declaration of Helsinki. Niigata university hospital review board approval was obtained (approval no: 2018-0403) and written informed consent was obtained from all patients.

### Data analysis

**Figure 2:**
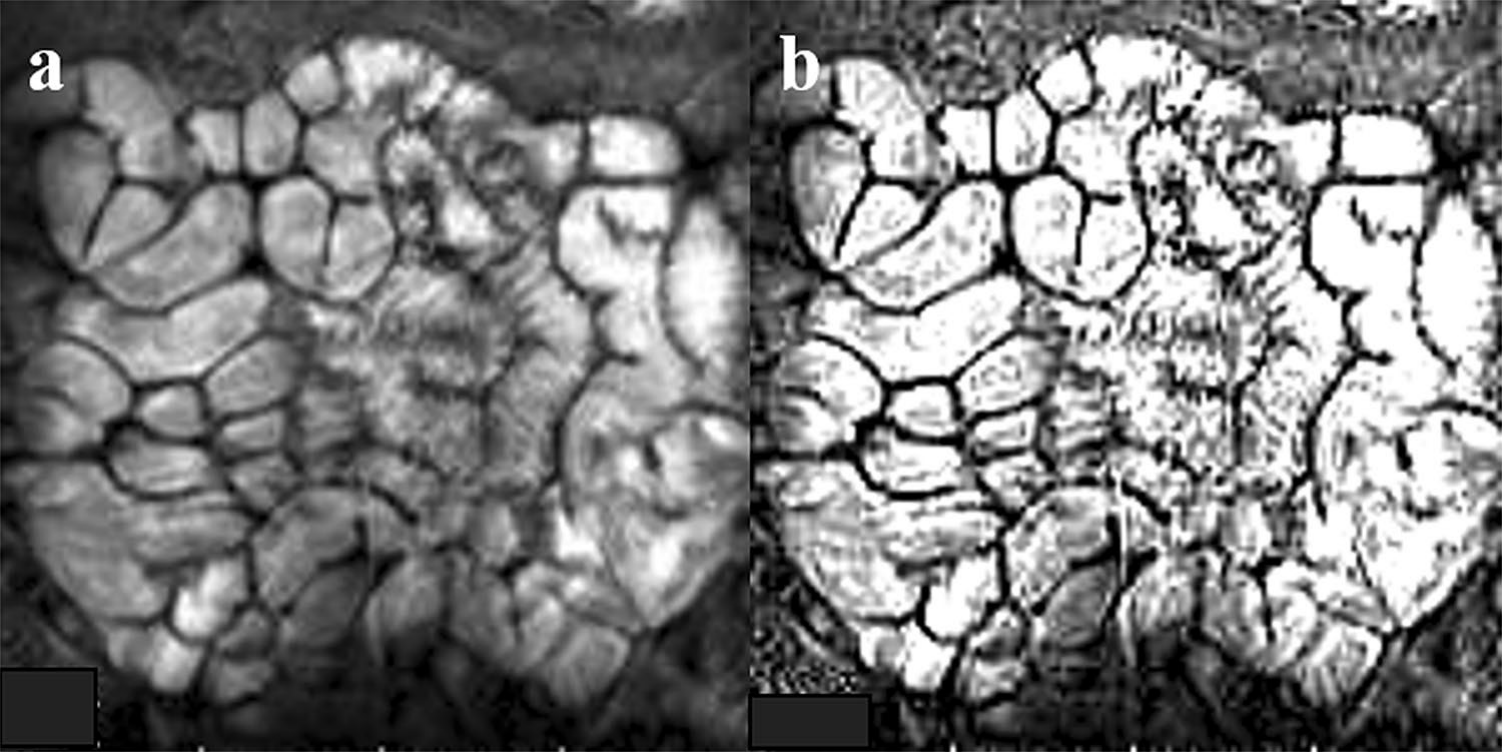
Edge of the small intestine can be enhanced automatically using software. Shown are (**a**) before enhancement and (**b**) afterward.

Cine MRI imaging analysis was performed using the Centricity DICOM Viewer software v. 2.2 (GE Medical Systems). An unsharp masking filter was used to improve the quality of all cine MRI scans degraded by noise (Fig. 2)^15^.

**Figure 3:**
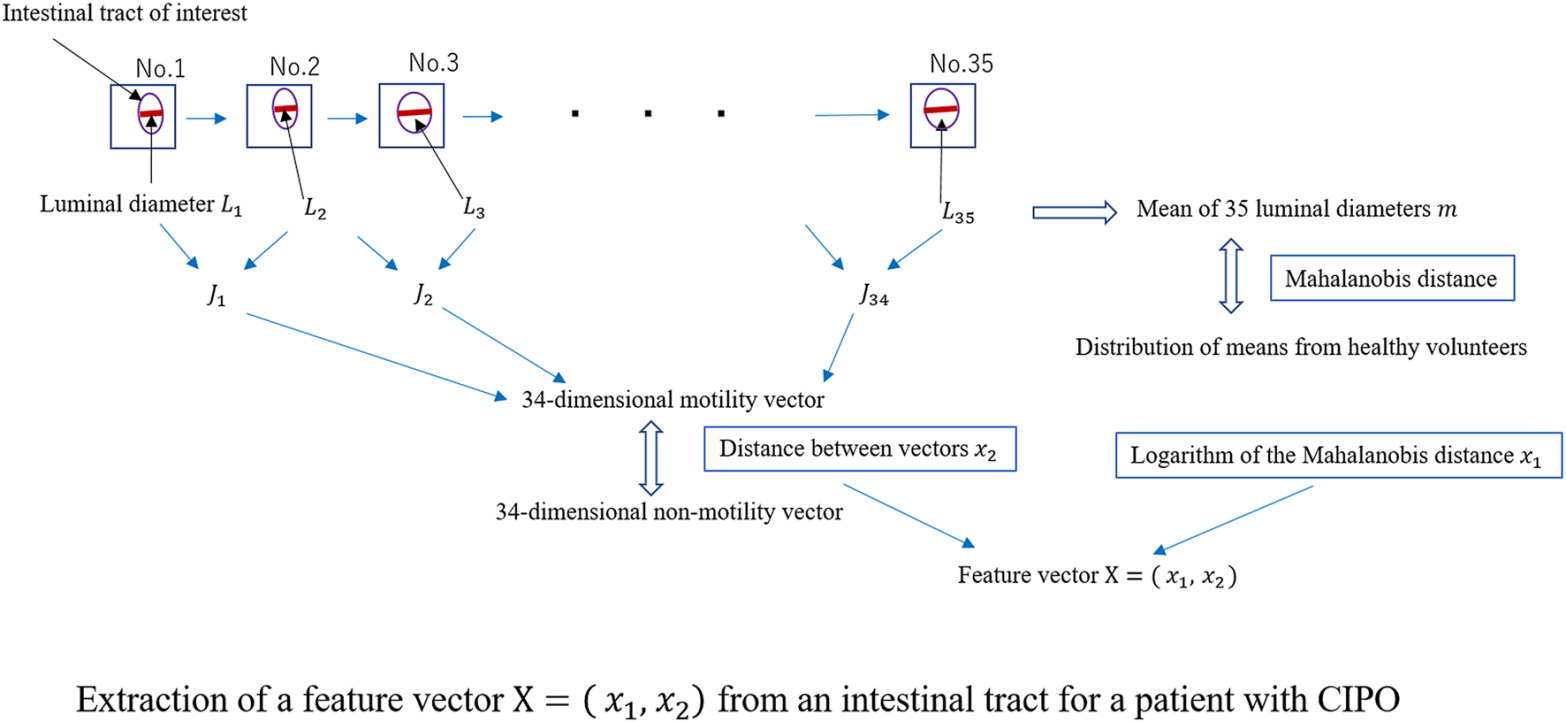
Process of extracting a two-dimensional feature vector from an intestinal tract of interest for a patient with chronic intestinal pseudo-obstruction in magnetic resonance imaging.

First, the observer marked the intestinal tracts, which sequentially appeared in all the 35 MRIs, and then visually selected a set of corresponding intestinal tracts from each of the 35 MRI images. The process of extracting a two-dimensional feature vector from the intestinal tract of interest for a patient with CIPO in MRI images is shown in Fig. 3. The intestinal tract of interest was represented as a two-dimensional feature vector and plotted on the two-dimensional feature space. We examined 78 intestinal tracts of interest from 18 samples. For each of the intestinal tracts of interest, 35 luminal diameters were separately measured. The total number of luminal diameters measured, which were used to calculate the J values and Mahalanobis distance, was 2730. With this method, it is important to consider inter-observer variability, because the variability is mainly produced by the observer. Hence, each luminal diameter was independently measured by two observers. Next, we checked the differences in the measurements performed by both observers and repeated the luminal diameter measurement in cases where a large difference was observed. The mean $m$ of the 35 luminal diameters was estimated. Similarly, the means from each of the healthy volunteers and the mean $\mu$ and variance ${\sigma }^{2}$ of the resulting means were also estimated. Next, the Mahalanobis distance was calculated, and its logarithm was obtained as the first feature x_1_. On the other hand, J values were calculated from luminal diameters, and the 34-dimensional motility vector was generated using J values. Then, the distance between the two vectors was adopted as the second feature, i.e., distance variation per time x_2_. The method is described in detail below.

**Figure 4:**
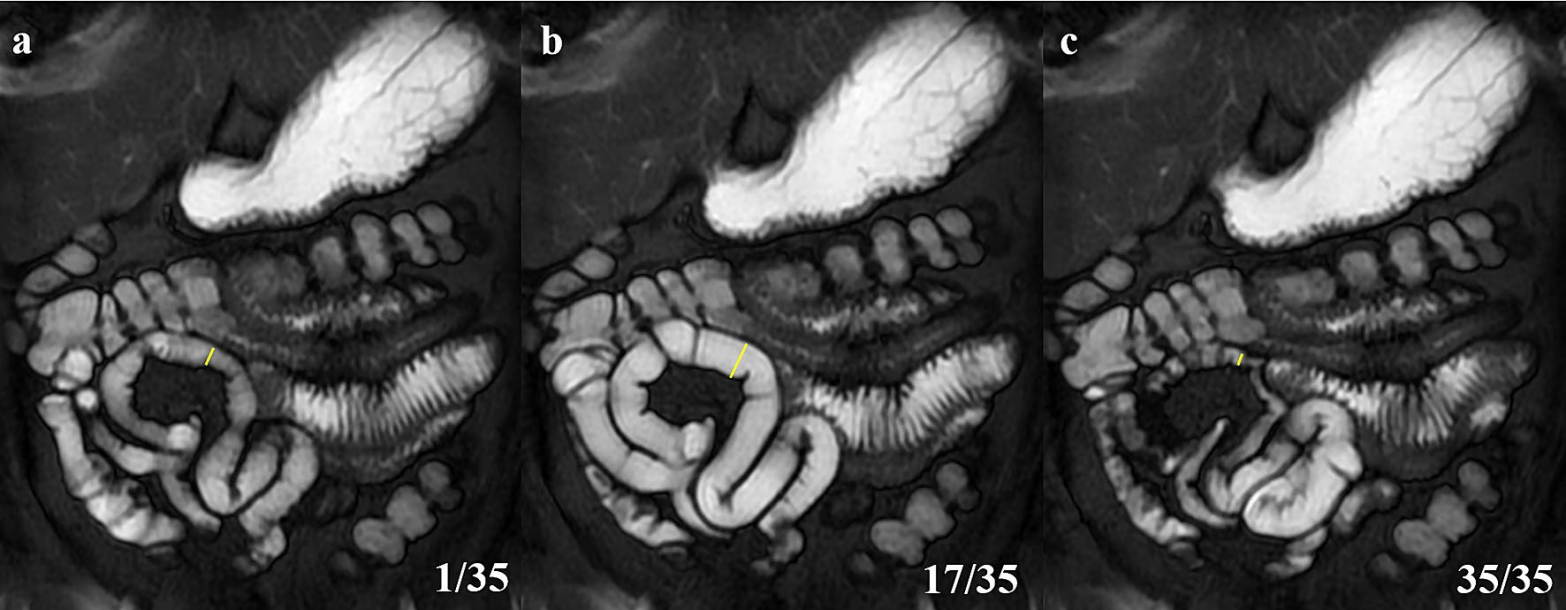
Luminal diameters of the small intestine (yellow line) are calculated using the software. This task was repeated for all sequential cine magnetic resonance imaging. The typical findings are shown as (**a**) (1st of the series), (b) (17th of the series), and (c) (35th of the series).

**Figure 5:**
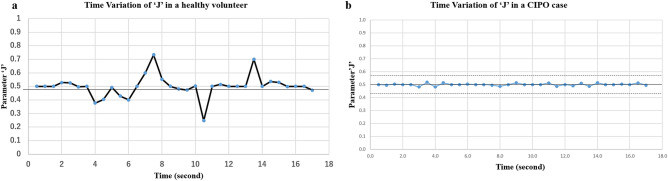
Time variation of parameter J in a healthy volunteer (**a**) and a case of chronic intestinal pseudo-obstruction (**b**).

At the corresponding location of the small intestinal tract, a line perpendicular to the long axis was drawn; this task was repeated for all sequential cine MRIs (Fig. 4). Using these diameters measured on 35 cine MRIs, the criterion ${\mathrm{J}}_{\mathrm{t}}$, which evaluates the motility of an intestinal tract, was defined by

1 $${\text{J}}_{{\text{t}}} = \frac{{{\text{L}}_{{\text{t}+1}} }}{{{\text{L}}_{{{\text{t}} }} + {\text{L}}_{{\text{t}+1}} }} \,\, t = 1, 2, \ldots , 34$$

where L_t_ is the luminal diameter at time t and L_t+1_ is that at time t+1. The J values ranged from 0 to 1. As the intestinal tract expands, the J value falls in the range of 0.5 ≨ J ≨ 1.0. Conversely, as the intestinal tract contracts, the value falls in the range of 0 ≨ J ≨ 0.5. Moreover, L_t_ = L_t+1_ leads to a J value of 0.5. Therefore, this value indicates that the intestinal tract has no motility (Fig. 5). Thus, by using the relative index calculated from sequentially adjacent images, we made the most of the motion characteristic of the cine MRI.

Next, we defined two 34-dimensional vectors; one was a motility vector whose components were the values of J, while the other was a non-motility vector whose components were all 0.5. The magnitude of the intestinal tract motility was determined by the distance between the motility and non-motility vectors; as motility increases, the distance lengthens. Moreover, the mean of 35 luminal diameters was estimated. We adopted the Mahalanobis distance^16^ to obtain the invariance to the scaling of the coordinate axes.

The Mahalanobis distance D(m) between the mean m of 35 luminal diameters of the intestinal tract of a patient and the volunteer distribution of means from healthy volunteers was defined as follows:

2 $$\mathrm{D}\left(m\right)=\frac{{\left(m-\mu \right)}^{2}}{{\sigma }^{2}}$$

where μ and σ^2^ are the sample mean and sample variance of the volunteer distribution, respectively. The sample mean and sample variance were estimated using the means for each of the intestinal tracts of healthy volunteers. Then, we calculated the logarithm of the Mahalanobis distance D(m); thus, any intestinal tract was represented as a two-dimensional vector whose components x_1_ and x_2_ were the logarithm of the Mahalanobis distance and distance variation per time, respectively. These x_1_ and x_2_ are new cine MRI parameters. In pattern recognition, the parameters x_1_ and x_2_ are called features. Each intestinal tract was represented as a feature vector in a two-dimensional feature space.

**Figure 6:**
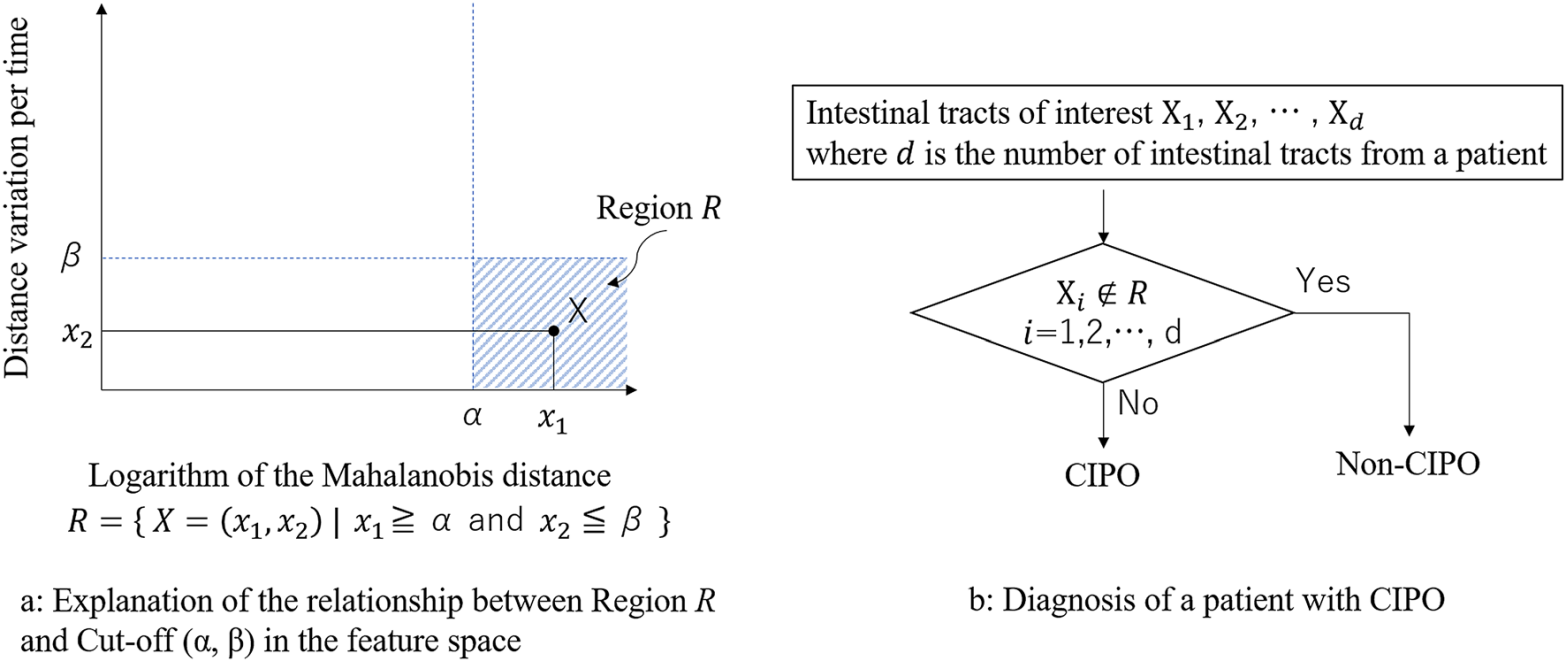
(**a**) Scheme of assessment of intestinal motility. It is deemed to have impaired motility if x_1_ ≥ α and x_2_ ≤ β, where α and β are the threshold values. An intestinal tract that had no impairment in motility satisfies the condition x_1_ < α or x_2_ > β, which implies denial of the proposition x_1_ ≥ α and x_2_ ≤ β. (**b**) The flowchart of cine-MRI diagnosis for chronic intestinal pseudo-obstruction (CIPO). A patient is deemed to have CIPO if any of the intestinal tracts with impaired motility was detected.

Once the intestinal tract of the patient was selected, it was deemed to have impaired motility if x_1_ ≥ α and x_2_ ≤ β, where α and β are the threshold values (mentioned below), as shown in Fig. 6a. An intestinal tract that had no impairment in motility satisfied the condition x_1_ < α or x_2_ > β, which implied denial of the proposition x_1_ ≥ α and x_2_ ≤ β. The patient was deemed to have CIPO if any of the intestinal tracts was shown to have impaired motility, as shown in Fig. 6b.

**Figure 7:**
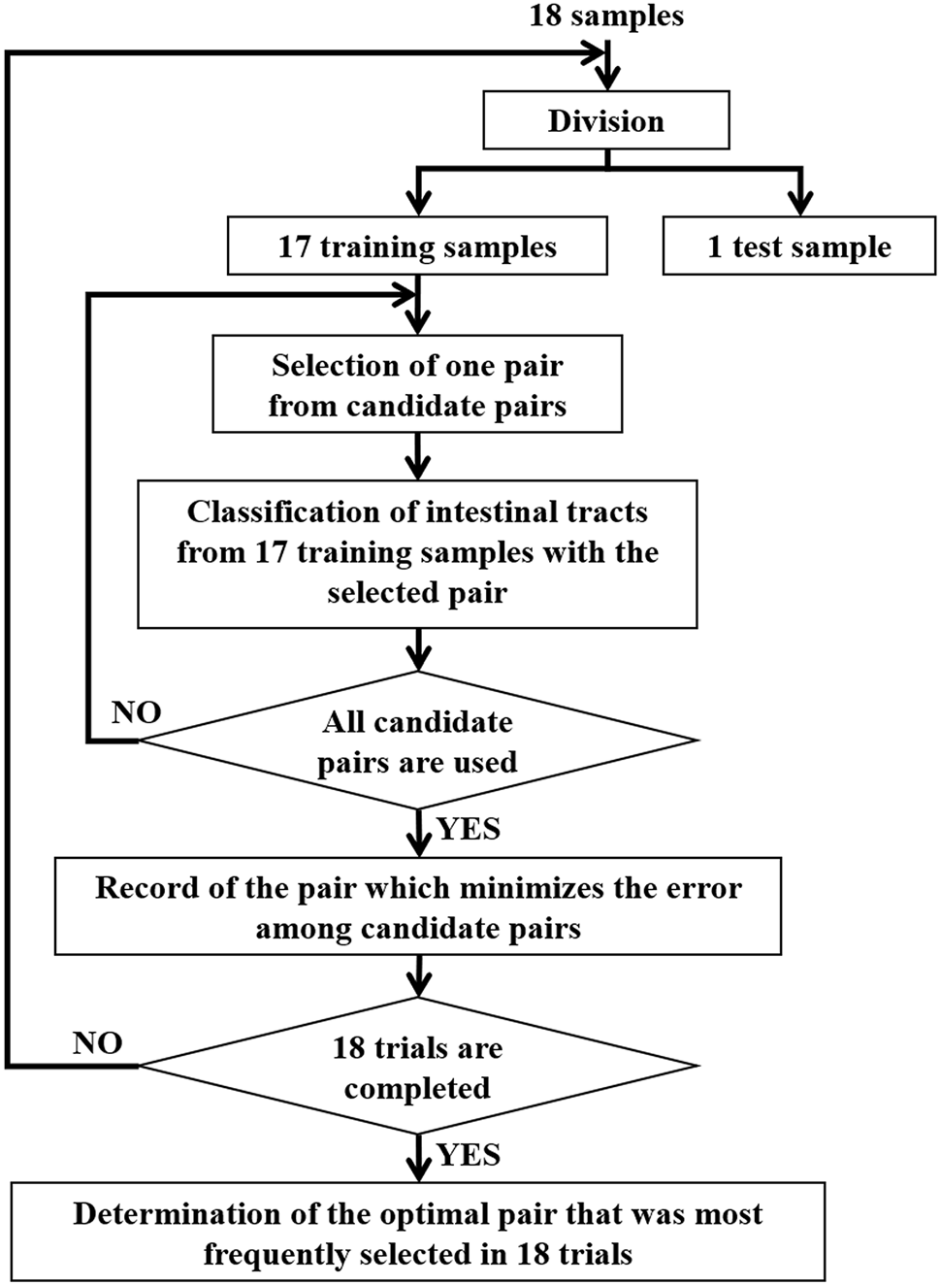
Scheme of optimization of threshold values.

As the values of α and β are critical for classifying individuals, we determined the optimal α and β pairing (i.e., that which minimized error) from among candidate pairs (Fig. 7). To determine the most robust pair that optimally takes into account the variability of samples, we used the leave-one-out method^17^, which generates artificial variability among the patients with the use of resampling^18^. There were 18 available samples; according to the leave-one-out method, one sample was excluded, and the remaining 17 were used as training samples. Note that the test sample was not used in optimization. Next, an α and β pair was selected from candidate pairs. Thereafter, we determined whether each intestinal tract of a training sample had severe impairment of motility using the selected α and β pair. If x_1_ ≥ α and x_2_ ≤ β, the intestinal tract was classified as an impaired one, and the number of misclassified intestinal tracts was counted from among intestinal tracts of 17 training samples to estimate the error. This was conducted for each candidate pair. Ultimately, we selected the pair that produced the lowest error among the intestinal tracts of 17 training samples. The above operation was repeated until each of the 18 samples had been excluded once. We determined the optimal pair that was most frequently selected from among the 18 trials.

### Statistics

Continuous variables, such as patient age and body mass index are presented as medians and interquartile ranges, while categorical variables such as sex are expressed as numbers and percentages. All statistical analyses were performed using EZR (Saitama Medical Center, Jichi Medical University, Saitama, Japan), which is a graphical user interface for R (The R Foundation for Statistical Computing, Vienna, Austria). More precisely, it is a modified version of R commander designed to add statistical functions frequently used in biostatistics^19^.

## Results

Seven patients with CIPO, including three men (42.9%) and four women (57.1%), whose age was 52 (38.5–54.5) years underwent cine MRI; their characteristics are shown in Table 1. The disease duration was 40.1 (36.6–118.8) months, and all the patients were underweight (body mass index = 17.3 [16.2–18.2] kg/m^2^) and had abdominal distention. Cine MRI examination was also performed on 11 healthy volunteers comprising six men (54.5%) and five women (45.5%) with an age of 35 (29.0–41.5) years.

**Table 1:** Clinical course and cine magnetic resonance imaging findings in patients with chronic intestinal obstruction.

Case No	Age (y)	Sex	BMI (kg/m^2^)	CIPO duration (months)	Symptoms^a^	Cine MRI		Treatment
						X_1_	X_2_	
1	37	M	18.2	180.3	D, V	1.79	0.22	Medication
2	52	M	19.4	35.7	D, V, P	2.68	0.10	Medication, Surgical bypass, HPN
3	61	M	17.1	6.5	D, V	1.12	0.16	Medication
4	55	F	18.2	81.0	D, V	2.79	0.04	Medication, surgical bypass, HPN
5	35	F	11.1	37.6	D, P, C	2.01	0.10	Surgical bypass
6	54	F	15	40.1	D, P	2.33	0.13	Surgical bypass
7	40	F	17.3	156.6	D, C	4.26	0.04	Medication

BMI, body mass index; CIPO, chronic intestinal obstruction; HPN, home parenteral nutrition; MRI, magnetic resonance imaging. ^a^D: abdominal distention, V: vomiting, P: Abdominal pain, C: constipation. ${\mathrm{x}}_{1}$: the logarithm of the Mahalanobis distance, ${\mathrm{x}}_{2}$: the distance variation per time.

### Threshold value for separating patients with CIPO from healthy volunteers

**Figure 8:**
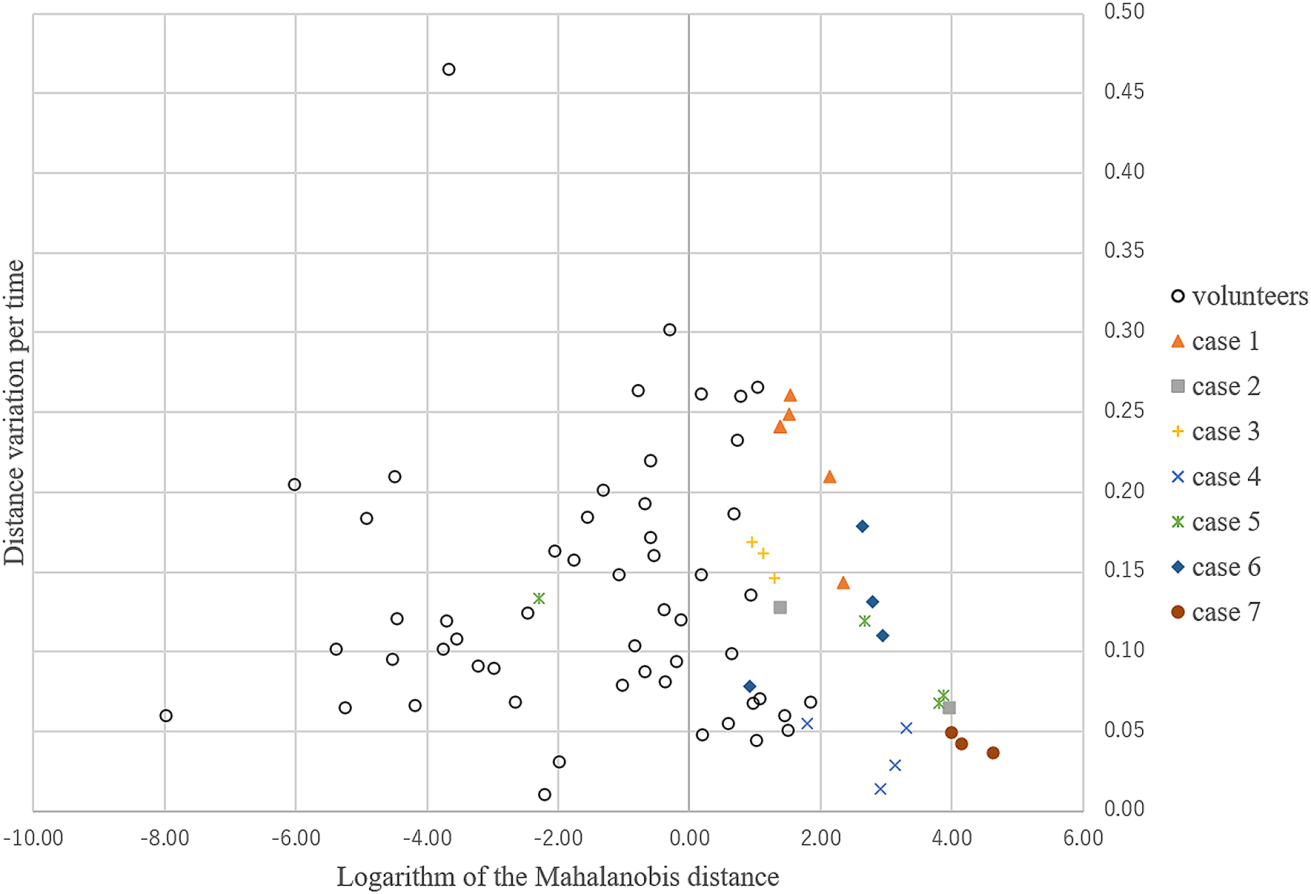
Scatter diagram of the logarithm of the Mahalanobis distance (horizontal axis) and distance variation per time (vertical axis) in patients with chronic intestinal pseudo-obstruction and healthy volunteers.

The logarithm of the Mahalanobis distance, as well as the distance variation per time, were calculated for each of the intestinal tracts from both patients with CIPO and healthy volunteers, and the data are shown graphically in Fig. 8 (Supplementary File S1).

**Figure 9:**
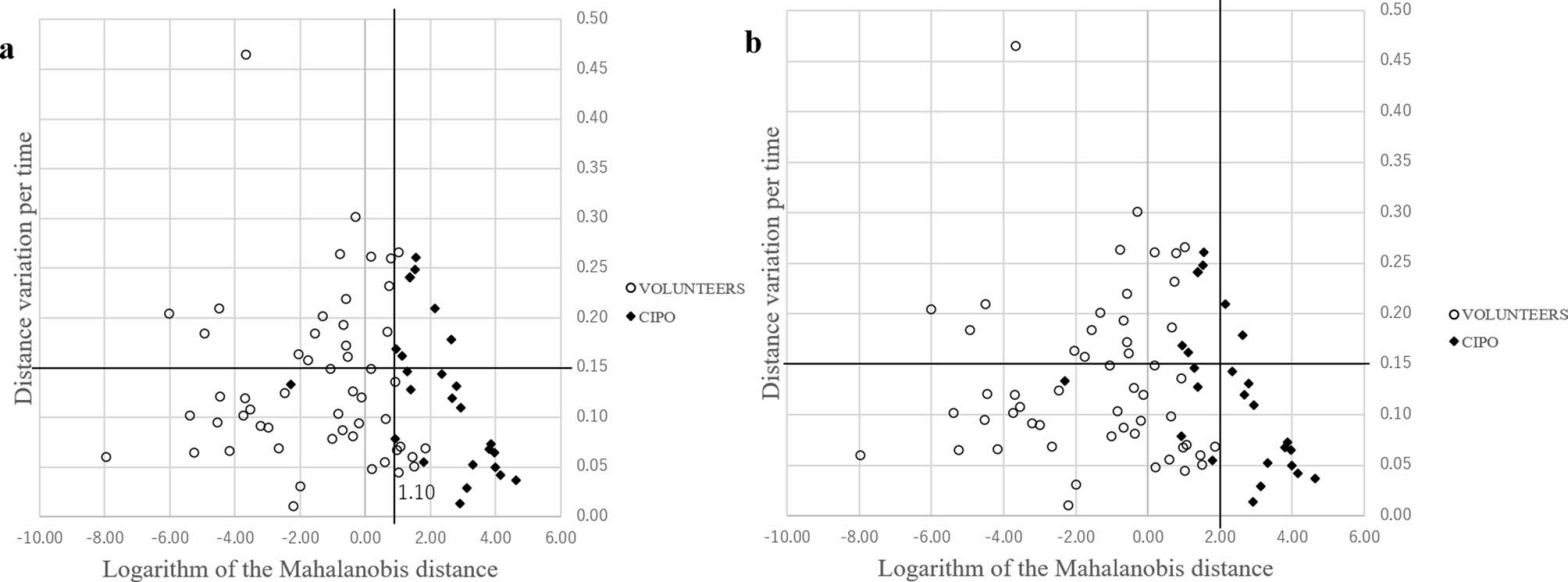
Scatter diagram for determining sensitivity and specificity in two-dimensional feature space. (**a**) α = 1.10 and β = 0.15 can be used to obtain the best sensitivity for chronic intestinal pseudo-obstruction (CIPO) diagnosis. (**b**) α = 2.00 and β = 0.15 can be used to obtain the maximum specificity for CIPO diagnosis.

The optimal pair of α = 1.10 and β = 0.15 was considered to be robust for the variability of samples. The parameters with these values produced a sensitivity of 1.00 (7/7) and specificity of 0.82 (9/11) (Fig. 9a); the error was 0.11 (2/18). On the other hand, a specificity of 1.00 (11/11) and sensitivity of 0.86 (6/7) were obtained when values of α = 2.00 and β = 0.15 were used (Fig. 9b).

### Severity of CIPO and cine MRI findings

**Figure 10:**
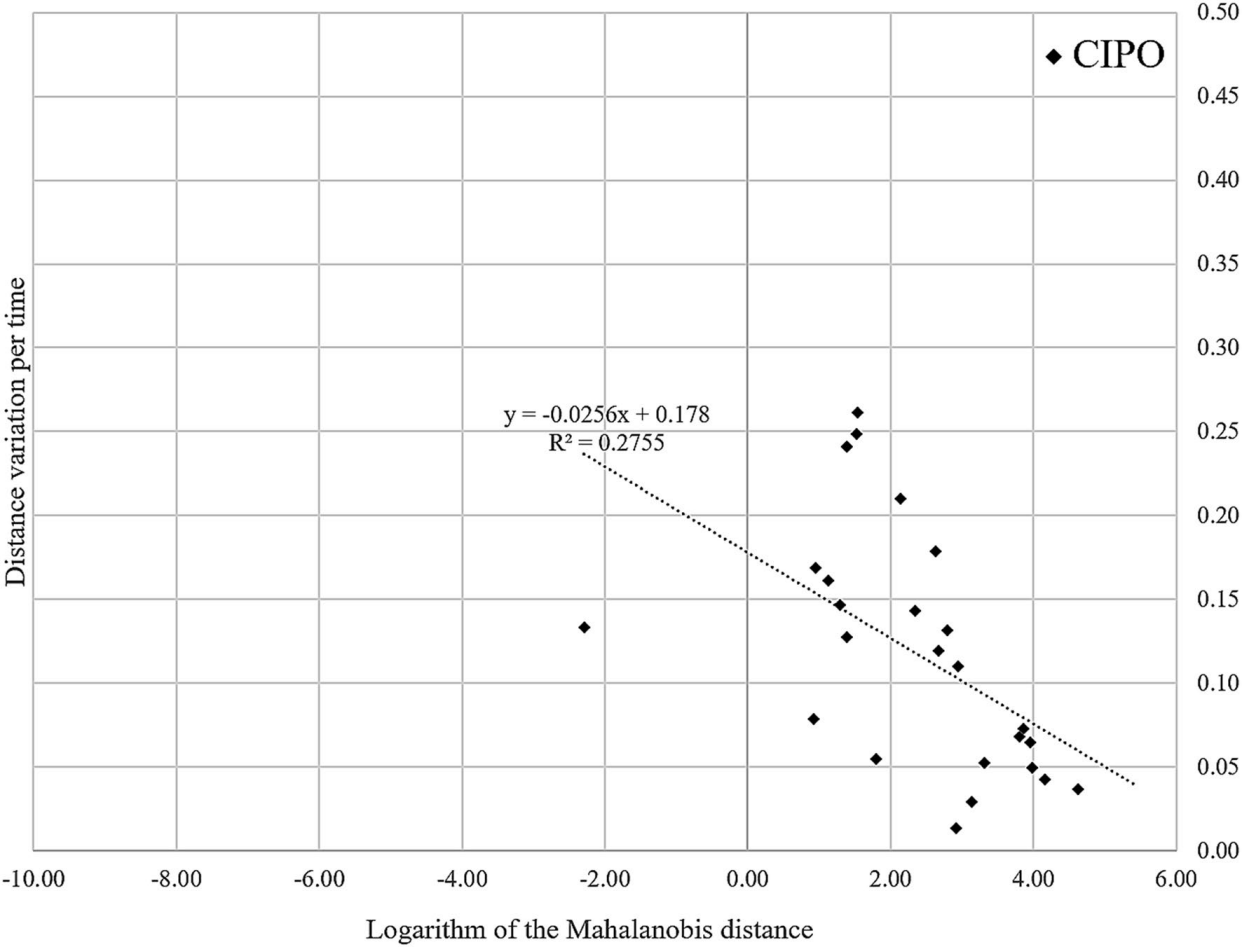
In patients with chronic intestinal obstruction, the logarithm of the Mahalanobis distance and distance variation per time were inversely correlated.

The severity of CIPO was determined based on the treatment required: mild CIPO cases were controlled by medication only; moderate cases required surgical intervention; and severe cases required permanent HPN (Table 1). The mean vector of the intestinal tract from each case was estimated as a representative characteristic. Table 1 shows the two components of the mean vector. Any of the intestinal tracts of severe cases belonged to the region defined by x_1_ ≥ 1.10 and x_2_ ≤ 0.15 in the two-dimensional feature space. Furthermore, in patients with CIPO, Pearson’s analysis of the logarithm of the Mahalanobis distance and the distance variation per time revealed a mild inverse correlation of − 0.52 (Fig. 10). When analyzing all participants, including patients and volunteers (n = 18) as well as volunteers alone (n = 11), no correlation was found (the Pearson’s correlation coefficients were − 0.15 and 0.01, respectively).

## Discussion

We successfully established cine MRI parameters that could different iate patients with CIPO from healthy volunteers. Our analysis also revealed that all patients with CIPO had impaired small intestinal motility. We assumed that for the intestinal tracts from a patient with CIPO, x_2_ became small, with the logarithm of the Mahalanobis distance x_1_ large. The results showed that the negative correlation between x_1_ and x_2_ was observed only for patients with CIPO.

We used the original parameters ‘logarithm of the Mahalanobis distance’ and ‘distance variation per time’ in this study because other previously reported parameters, such as intestinal ‘amplitude’ and ‘contraction cycle’ were difficult to determine during our previous efforts. This was presumably because of the various types of small intestinal contraction that occur concurrently, including peristalsis, segmentation, and phase III migrating motor complex^8,20^. This makes it difficult to distinguish the type of each contraction definitively; moreover, the ‘amplitude’ and ‘contraction cycle’ are not considered single or regular. Instead, in this study, bowel contraction or expansion was evaluated by J < 0.5 or J > 0.5, which just focus on the rate of variability per time. Given that luminal diameters of patients with CIPO have diagnostic significance only when they are compared with those of healthy controls, the Mahalanobis distance was thought to be a proper metric in evaluating luminal diameters, since it normalized the luminal diameter of patients with CIPO using that of healthy volunteers. Furthermore, the other advantage of using the Mahalanobis distance is that it is not affected by the scan resolutions and can be applied to scans from different types of MRI equipment, which is different from the Euclidean distance (Supplementary File S2).

Although the feature vectors of healthy volunteers had widely distributed values in this study, the threshold value of 1.10 for the parameter x_1_ was found to be satisfactory for CIPO diagnosis. Furthermore, the luminal diameters were selected for analysis from all parts of the small intestine because we could not always measure these diameters in specific areas (i.e., the proximal jejunum, middle intestine, and distal ileum) given that the analyses were performed on sliced views, wherein certain parts of the small intestine were often in a blind spot and thus invisible. Another drawback was that the intestines sliced horizontally or perpendicularly were not always captured given the small intestine’s tortuousness in the three-dimensional abdominal cavity.

A key finding in this study was that all patients with CIPO had impaired small intestinal motility. However, in some cases, lower colonic propulsion may have been impaired, which further inhibited small bowel propagation. Furthermore, the severity of CIPO was reflected in the cine MRI findings. Therefore, our calculated parameters may potentially be useful for clinical decision-making. In contrast, the parameters calculated for healthy volunteers had a wide range, which was a notable observation. Further studies of cine MRI are required to reliably differentiate CIPO from other disorders, such as irritable bowel syndrome, although determining the definitive parameters is likely to be challenging. At a minimum, longer cine MRI observation is necessary, and the extent of small intestinal contraction (peristalsis, segmentation, and migrating motor complex), should be analyzed.

Based on our results, using cine MRI together with computer-assisted or artificial intelligence-driven diagnosis of gastrointestinal motility may have potential to accurately identify patients with CIPO, although several drawbacks should be addressed (such as the limitations of sliced-based analysis). Although there was a bias in the selection of the intestinal tract and positioning of the luminal diameter, we adopted the effective method of visually measuring only one measurement point, i.e., one luminal diameter for each of the corresponding intestinal tracts. Our study focused on small intestinal motility, and colonic motility should be further explored in additional investigations. The small intestine usually functions to move and clear its luminal contents before more food is ingested. In contrast, the colon is idle up to 90% of the time. Hence, cine MRI may be unsuitable for the evaluation of colonic movement given its glacial pace.

Our study had several limitations that should be acknowledged. First, only a small number of patients with CIPO were analyzed because of the rarity of this disorder. Therefore, data analysis between CIPO and healthy volunteers was limited. Classification experiments were conducted using the α and β values optimized by the leave-one-out method with 18 samples. The performance should be evaluated for independent test samples. However, this remains one of the issues to be studied further. Second, we classified the severity of CIPO based on the required treatment; however, severity is determined using a complicated scale in real-world clinical settings. For example, patient 7 had severe symptoms that were controlled with many medications and well-balanced meals. Third, the backgrounds of patients with CIPO and healthy volunteers, such as age, sex, and body mass index were different, which may also have affected intestinal motility.

In conclusion, our new cine MRI parameters have the potential to assist in the differential diagnosis of patients suspected of having CIPO, including the severity thereof. The wide ranges of distributions for feature vectors of healthy volunteers may reflect the complicated function of the small intestine, which should be elucidated using additional cine MRI studies in the future.

## Supplementary Information

Supplementary Information 1.

Supplementary Information 2.

## Data Availability

All data used in the study are already provided in the tables, figures, and online supplementary materials.
