# Supplementary material for: New cine magnetic resonance imaging parameters for the differential diagnosis of chronic intestinal pseudo-obstruction
Source: Sci Rep. 2021 Nov 26;11:22974. doi: 10.1038/s41598-021-02268-1 (PMC8626471; doi:10.1038/s41598-021-02268-1)
Supplement: Supplementary file 1 — Supplementary Information 1. [file 41598_2021_2268_MOESM1_ESM.docx]

**Supplemental file 1:**

| Case No. | Intestine No. | $\mathbf{x}_{\boldsymbol{1}}$: Logarithm of the Mahalanobis distance | $\mathbf{x}_{\boldsymbol{2}}$:Distance variation per time |
| --- | --- | --- | --- |
| case 1 | 1 | 1.53 | 0.25 |
|  | 2 | 2.14 | 0.21 |
|  | 3 | 2.35 | 0.14 |
|  | 4 | 1.54 | 0.26 |
|  | 5 | 1.39 | 0.24 |
| case 2 | 1 | 3.96 | 0.06 |
|  | 2 | 1.39 | 0.13 |
| case 3 | 1 | 1.29 | 0.15 |
|  | 2 | 0.95 | 0.17 |
|  | 3 | 1.12 | 0.16 |
| case 4 | 1 | 3.31 | 0.05 |
|  | 2 | 1.80 | 0.06 |
|  | 3 | 2.92 | 0.01 |
|  | 4 | 3.13 | 0.03 |
| case 5 | 1 | 2.67 | 0.12 |
|  | 2 | -2.29 | 0.13 |
|  | 3 | 3.87 | 0.07 |
|  | 4 | 3.80 | 0.07 |
| case 6 | 1 | 0.93 | 0.08 |
|  | 2 | 2.80 | 0.13 |
|  | 3 | 2.63 | 0.18 |
|  | 4 | 2.94 | 0.11 |
| case 7 | 1 | 3.99 | 0.05 |
|  | 2 | 4.15 | 0.04 |
|  | 3 | 4.63 | 0.04 |
| Volunteer 1 | 1 | -1.02 | 0.08 |
|  | 2 | -3.70 | 0.12 |
|  | 3 | -2.21 | 0.01 |
|  | 4 | -3.22 | 0.09 |
| Volunteer 2 | 1 | -4.18 | 0.07 |
|  | 2 | -0.12 | 0.12 |
|  | 3 | -3.74 | 0.10 |
|  | 4 | -2.66 | 0.07 |
|  | 5 | -0.39 | 0.13 |
| Volunteer 3 | 1 | -4.49 | 0.21 |
|  | 2 | -0.69 | 0.09 |
|  | 3 | -1.06 | 0.15 |
|  | 4 | 0.97 | 0.07 |
| Volunteer 4 | 1 | -2.98 | 0.09 |
|  | 2 | 0.78 | 0.26 |
|  | 3 | -3.67 | 0.46 |
|  | 4 | -0.77 | 0.26 |
|  | 5 | -0.30 | 0.30 |
| Volunteer 5 | 1 | -0.53 | 0.16 |
|  | 2 | -2.47 | 0.12 |
|  | 3 | -5.38 | 0.10 |
|  | 4 | 0.19 | 0.15 |
|  | 5 | -1.75 | 0.16 |
| Volunteer 6 | 1 | -0.37 | 0.08 |
|  | 2 | -4.45 | 0.12 |
|  | 3 | -0.59 | 0.17 |
|  | 4 | -0.83 | 0.10 |
|  | 5 | 0.19 | 0.26 |
| Volunteer 7 | 1 | -4.53 | 0.10 |
|  | 2 | -3.54 | 0.11 |
|  | 3 | 1.07 | 0.07 |
|  | 4 | 0.64 | 0.10 |
|  | 5 | -0.68 | 0.19 |
| Volunteer 8 | 1 | -0.59 | 0.22 |
|  | 2 | -4.93 | 0.18 |
|  | 3 | -7.97 | 0.06 |
|  | 4 | -5.25 | 0.06 |
|  | 5 | 1.51 | 0.05 |
| Volunteer 9 | 1 | 0.67 | 0.19 |
|  | 2 | 0.93 | 0.14 |
|  | 3 | -2.05 | 0.16 |
|  | 4 | 0.73 | 0.23 |
|  | 5 | 0.20 | 0.05 |
| Volunteer 10 | 1 | 1.02 | 0.04 |
|  | 2 | -1.55 | 0.18 |
|  | 3 | 1.46 | 0.06 |
|  | 4 | -0.19 | 0.09 |
|  | 5 | 1.85 | 0.07 |
| Volunteer 11 | 1 | -6.01 | 0.20 |
|  | 2 | -1.31 | 0.20 |
|  | 3 | 1.04 | 0.27 |
|  | 4 | -1.98 | 0.03 |
|  | 5 | 0.59 | 0.06 |
