# Supplementary figures and images for: New cine magnetic resonance imaging parameters for the differential diagnosis of chronic intestinal pseudo-obstruction
Source: Sci Rep. 2021 Nov 26;11:22974. doi: 10.1038/s41598-021-02268-1 (PMC8626471; doi:10.1038/s41598-021-02268-1)

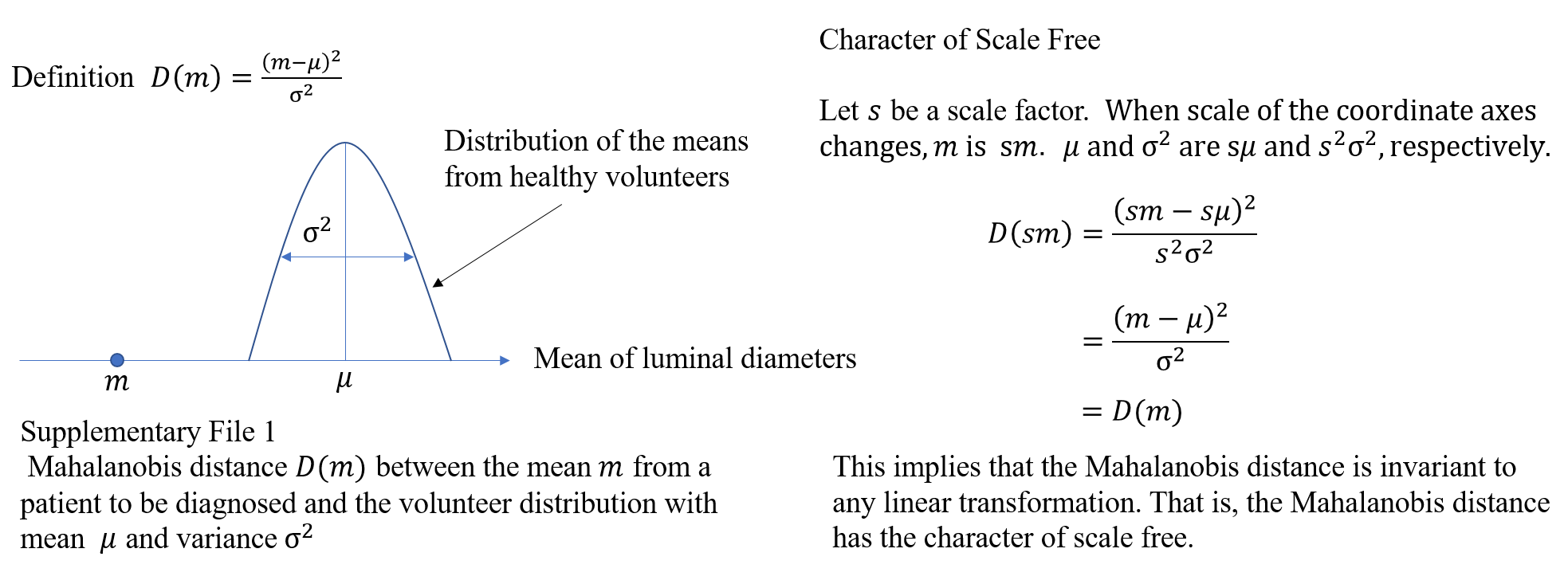

Supplement: Supplementary file 2 — Supplementary Information 2. [file 41598_2021_2268_MOESM2_ESM.tif]
